# Norcantharidin induces growth inhibition and apoptosis of glioma cells by blocking the Raf/MEK/ERK pathway

**DOI:** 10.1186/1477-7819-12-207

**Published:** 2014-07-15

**Authors:** Jie Zheng, Wei Du, Lai-jun Song, Rui Zhang, Lai-guang Sun, Feng-guo Chen, Xin-ting Wei

**Affiliations:** 1 Department of Neurosurgery, Xinxiang Central Hospital, 56 Jinsui Avenue, Xinxiang, Henan, China; 2 Department of Neurosurgery, the First Affiliated Hospital of Zhengzhou University, Zhengzhou, Henan, China; 3 Department of Respiratory Medicine, Xinxiang Central Hospital, Xinxiang, Henan, China

**Keywords:** Glioma, Norcantharidin, MAPK, Apoptosis

## Abstract

**Background:**

Malignant gliomas represent the most common primary brain tumors. The prognosis of patients with malignant gliomas is poor in spite of current intensive therapy and novel therapeutic modalities are needed. Here we report that norcantharidin is effective in growth inhibition of glioma cell lines *in vitro*.

**Methods:**

Glioma cell lines (U87 and C6) were treated with norcantharidin. The effects of norcantharidin on the proliferation and apoptosis of glioma cells were measured by 3-[4,5-dimethylthiazol-2-thiazolyl]-2,5-diphenyl-tetrazolium bromide (MTT) assay and flow cytometry. Western blotting was employed to determine the signaling pathway changes.

**Results:**

The results showed that norcantharidin effectively inhibited cell growth and induced apoptosis in glioma cells, which was concurrent with inhibition of the expression of phospho-MEK and phospho-ERK. Furthermore, the expression anti-apoptotic proteins Bcl-2 and Mcl-1 significantly reduced, but no changes in Bcl-xL and Bax.

**Conclusions:**

Our findings demonstrate that norcantharidin is effective for growth inhibition of glioma cell lines and suggest that norcantharidin may be a new therapeutic option for patients with glioma.

## Background

Malignant gliomas are the most common primary tumors of the central nervous system in adults. Surgical resection with adjuvant radiotherapy and temozolomide-based chemotherapy are the mainstays of treatment [1]. Despite tremendous efforts in diagnosis and therapeutic strategies, the clinical outcome remains dismaying. Because of the infiltrative growth of gliomas, it is difficult to resect the whole tumor without causing serious damage of the brain. Therefore, innovative treatment approaches are urgently needed [2,3].

Recent publications indicate that the majority of gliomas display upregulated Raf/MEK/extracellular signal-regulated kinase (ERK) pathway, which is an essential serine/threonine kinase constituent of the mitogen-activated protein kinase (MAPK) pathway. Upon activation by growth factors, serum, cytokines, and osmotic stresses, ERK can phosphorylate and regulate multiple substrates such as cytoskeletal proteins, kinases, and transcription factors. These events in turn result in gene expression changes and regulate many fundamental cellular functions such as cell growth, proliferation, and apoptosis. The upregulation of the Raf/MEK/ERK pathway has been proven to take part in the amplification of mitogenic stimuli and promotion of cellular proliferation of malignant gliomas [4-6]. Therefore downregulation of the Raf/MEK/ERK pathway may represent appropriate alternate therapy for glioma patients [7].

Cantharidin (exo-2,3-dimethyl-7-oxabicyclo-, hep-tane-2,3-dicarb-oxylic acidanhydride) is a bioactive compound, purified from mylabris, the dried body of the Chinese blister beetle. Because of severe nephrotoxicity and inflammatory side effects, CTD has been modified to a demethylated form, norcantharidin (NCTD) [8]. Moreover, many studies have reported that NCTD could exert its anti-tumor characteristics on various cancer cells such as bladder carcinoma, leukemia, colorectal carcinoma, hepatoma, and medulloblastoma [9-13]. Currently, NCTD has been reported to induce apoptosis in DAOY and UW228 medulloblastoma cells through inhibition of Wnt/β-catenin signaling activation [13]. However, the molecular mechanisms are still not well understood.

In the present study, we attempt to investigate the growth inhibition effect and the anti-cancer mechanism of NCTD in the glioma cell lines U87 and C6. Our results suggested that Bcl-2 family proteins participated in the apoptosis induced by NCTD. In addition, it was revealed that NCTD may exert its anti-cancer activity through the suppression of the Raf/MEK/ERK pathway.

## Methods

### Cells and reagents

Glioma cell lines U87 and C6 were purchased from American Type Culture Collection (Manassas, VA, USA). All cell lines were maintained in Dulbecco’s Modified Eagle Medium (DMEM) supplemented with 10% fetal bovine serum (FBS) and P/S solution (10,000 U/mL penicillin, 10,000 μg/mL sreptomycin) at 37°C and 5% CO_2_. NCTD was purchased from Sigma Chemical (St Louis, MO, USA) and dissolved in dimethyl sulfoxide (DMSO).

### MTT viability assay

Cell viability was measured by 3-[4,5-dimethylthiazol-2-thiazolyl]-2,5-diphenyl- tetrazolium bromide (MTT) assay. Glioma cells were seeded in 96-well culture clusters (Corning, NY, USA) at a density of 5,000 to 6,000 cells/well in 100 μL medium and incubated at 37°C in a humidified incubator for 24 h. On the following day, cells were treated with desired concentrations of NCTD. Four hours before desired time points, 10 μL of 10 mg/mL MTT was added. After incubation for 4 h, the plates were depleted and 200 μL DMSO was added to each well, and viable cells detected by measuring absorbance at 570 nm using MRX II absorbance reader (DYNEX Technologies, Chantilly, VA, USA). The cell viability was expressed as the percentage of absorbance in cells with NCTD treatment *versus* the control group. All experimental concentrations were replicated in triplicate. Results were expressed as means ± SD values.

### Apoptosis analysis by flow cytometer

Apoptotic cells were measured with Annexin V/FITC kit (BD Biosciences, Sparks, MD, USA) according to the manufacturer’s instructions. Cells were cultured in six-well plates at 3 × 10^5^ cells per well and treated with the agents for 10 h. The cells were harvested, washed with cold PBS, and then resuspended in 500 μL of binding buffer. A total of 5 μL of annexinV-FITC solution and 10 μL PI (1 g/mL) were added to these cells. Cells were then incubated in the dark for 30 min at room temperature prior to analysis by flow cytometry. Using flow cytometer (Beckman Coulter FC500, USA) to detect apoptosis through channels two and three. In all, 10,000 cells were collected for each sample.

### Western blotting analysis

Cells were plated in tissue culture dishes overnight and treated with different concentrations of NCTD for 24 h. After harvest and washout with new fresh culture medium, the cells were resuspended in lysis buffer containing protease inhibitor cocktail (Amresco, Solon, OH, USA). Equal amount of total protein extracts were separated by 10% standard sodium dodecyl sulfate polyacrylamide gel electrophoresis (SDS-PAGE) and transferred onto a polyvinylidene fluoride (PVDF) membrane (0.45 mm, Millipore, Bedford, MA, USA). Membranes were blocked with 5% fat-free milk and 0.1% Tween-20 in Tris-buffered saline, then incubated with the following primary antibodies as follows: MEK, ERK, phospho-MEK, phospho-ERK, Bcl-2, Bcl-xL, Mcl-1, Bax, and GAPDH (Cell Signaling Technology). Horseradish peroxidase-linked anti-mouse or anti-rabbit IgG were then used as secondary antibody, followed by detection by enhanced chemiluminescence (Amersham Bioscience, Piscataway, NJ, USA).

### Statistical analysis

Data were expressed as means ± standard deviation (SD). Statistical analysis was done using one-way analysis of variance (ANOVA) via SPSS 13.0 software (SPSS, Chicago, IL, USA). A value of *P* <0.05 was considered statistically significant.

## Results

### Cell growth inhibition of NCTD on glioma cells

**Figure 1:**
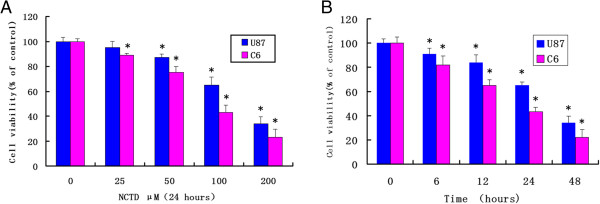
**Dose- and time-dependent inhibition of proliferation of glioma cells by NCTD treatment. (A)** U87 and C6 cell lines were treated with various doses of NCTD for 24 h. **(B)** The cells were treated by 100 μM NCTD for various time periods. At the end of incubation, the cell survival rates were determined by MTT methods. Cell viability was expressed as the percentage of cell survival compared with the control. Data were from three independent experiments. *P <0.05 compared to the control group.

In order to investigate the effect of NCTD on inhibition of proliferation of glioma, we exposed U87 and C6 cells to drug from 25 to 200 μM for desired time point. MTT assays demonstrated that NCTD exerted a dose- and time-dependent cell growth inhibition of U87 and C6 cells. By 24 h, the average IC50s for NCTD of U87 and C6 cells were 123.2 μM and 91.3 μM, respectively (Figure 1).

### NCTD causes glioma cell apoptosis

**Figure 2:**
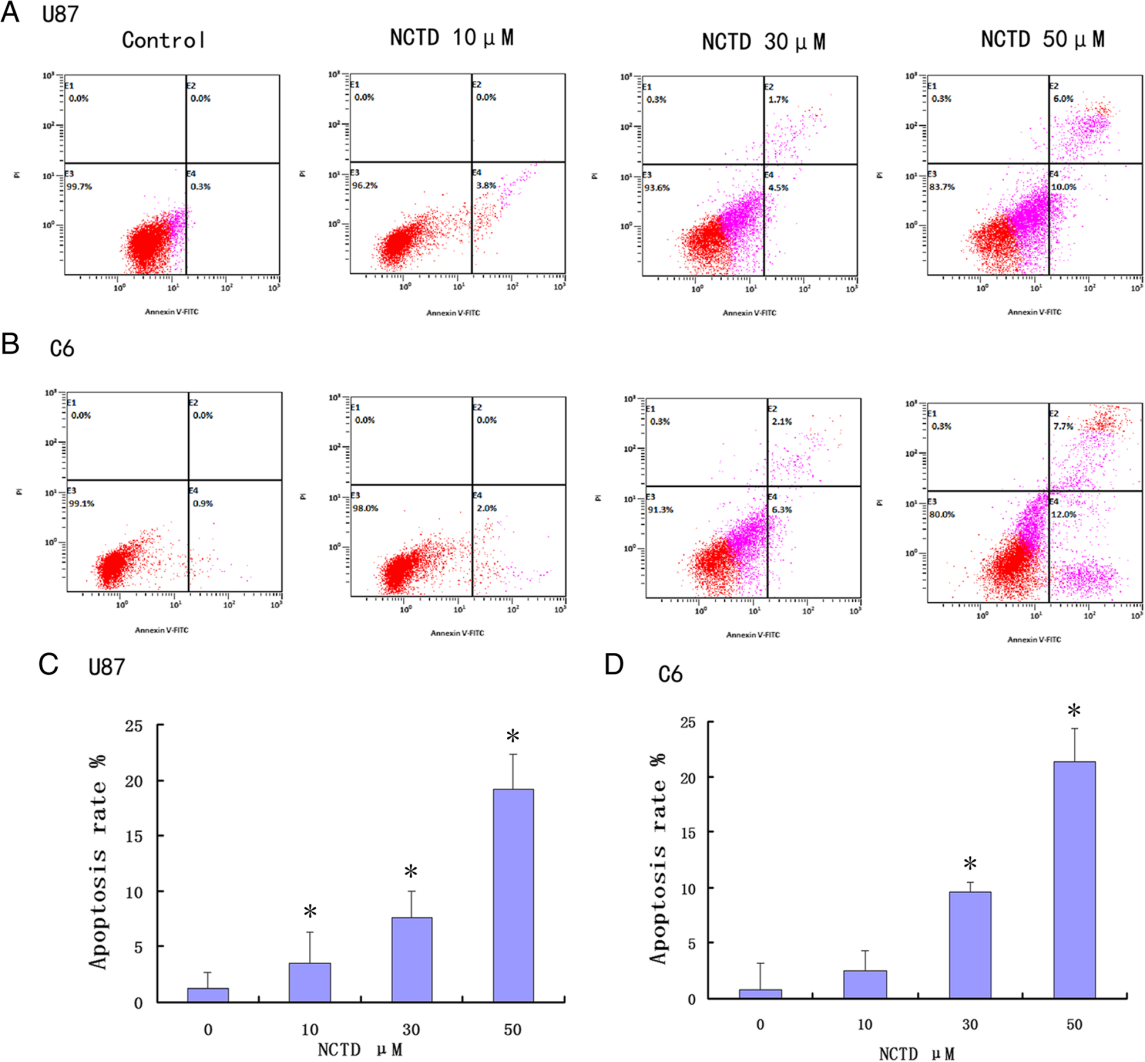
**NCTD caused apoptotic death in U87 (A, C) and C6 (B, D) cells.** Following 10 h of cell treatments, cells were collected and stained with Annexin V/PI followed by FACS analysis. Representative FACS analysis scatter-grams of Annexin V/PI stained 0, 10, 30, and 50 μM NCTD treatment showed four different cell populations marked as: double negative (unstained) cells showing live cell population (lower left), Annexin V positive and PI negative stained cells showing early apoptosis (lower right), Annexin V/PI double-stained cells showing late apoptosis (upper right), and finally PI positive and Annexin V negative stained cells showing dead cells (upper left). Apoptosis was defined as Annexin V staining positive. *P <0.05 compared to the control group.

In our assay, apoptotic death assay employing Annexin V/PI staining followed by fluorescent activated cell sorter (FACS) analysis clearly showed apoptotic effect of NCTD on glioma cells. As shown in Figure 2, the four quadrants in each panel correspond, respectively, to: necrotic cells (upper left), apoptotic late cells (upper right), apoptotic early cells (lower right), viable cells (lower left). Ten hours after treatment with NCTD, the results confirmed a dose-dependent apoptotic effect of NCTD on glioma cells.

### NCTD inhibits Raf/MEK/ERK signaling pathway in glioma cells

**Figure 3:**
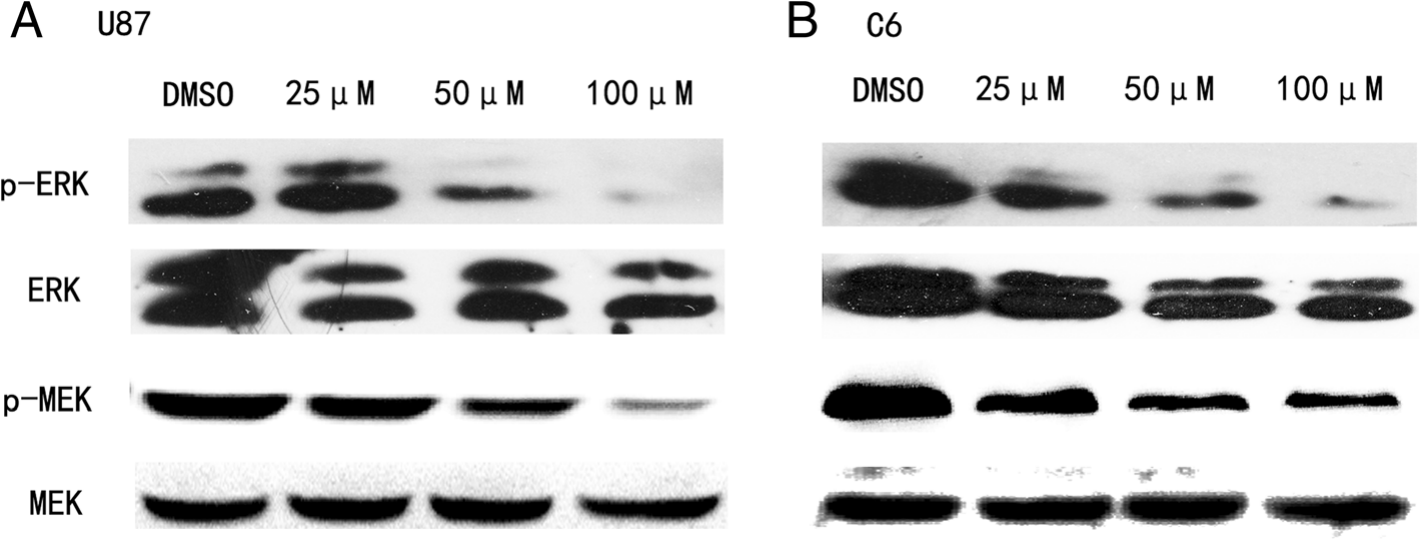
**NCTD inhibits Raf/MEK/ERK signaling pathway in U87 (A) and C6 (B) cells.** Cells were treated with the indicated concentrations of NCTD for 24 h. After treatment, whole cell protein extracts were prepared, and equal amounts of total protein were resolved on SDS-PAGE gels. Western blotting analysis was performed using specific antibodies against the indicated proteins.

The Raf/MEK/ERK pathway is downstream of Ras activation, and tyrosine phosphorylation of these proteins is essential for cancer cell proliferation. To correlate growth inhibition and apoptotic induction with NCTD therapy, we evaluated the effect of NCTD on the phosphorylation of these proteins by western blotting. We compared the phosphorylation of these proteins in cells treated with various concentrations of NCTD for 24 h. As shown in Figure 3, the results of western blotting showed that NCTD inhibited p-MEK and p-ERK dose-dependently (Figure 3).

### Effect of NCTD on the levels of pro-apoptotic protein Bax, anti-apoptotic proteins Bcl-2, Bcl-xL, and Mcl-1

**Figure 4:**
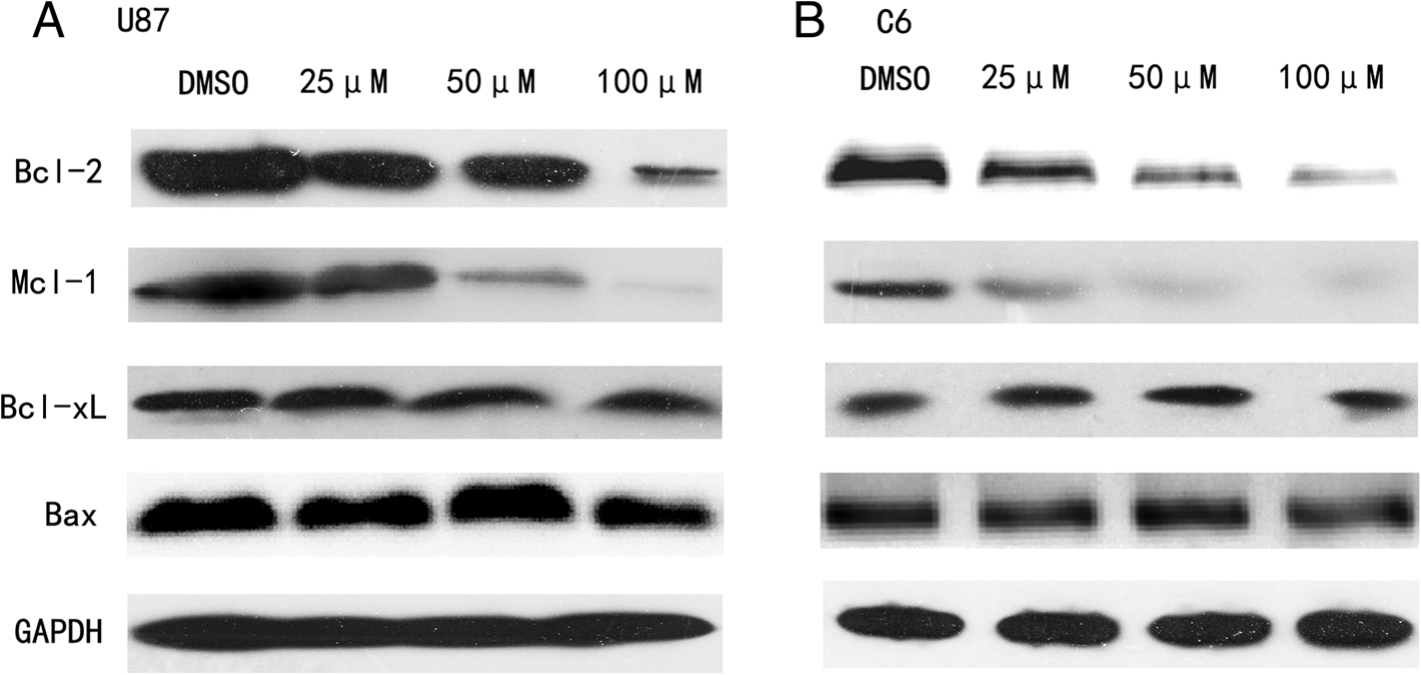
**NCTD downregulates Bcl-2 and Mcl-1 levels in U87 (A) and C6 (B) cells, but has little effect on expression of Bcl-xL or Bax.** The cells were harvested after incubation with the indicated concentrations of NCTD for 24 h. GAPDH was used as an internal control.

Because the intrinsic pathway of apoptosis is controlled by Bcl-2 family proteins, and cell death decisions are regulated by the balance between proapoptotic (for example, Bax) and antiapoptotic (for example, Bcl-2, Bcl-xL, Mcl-1, and so on) proteins. We also detected the effect of agent treatment on such proteins. As shown in Figure 4, NCTD–treated glioma cells for 24 h had little or no effect on expression of Bcl-xL or Bax. However, treatment of U87 and C6 cells with NCTD resulted in a significant downregulation of Bcl-2 and Mcl-1. Together, this analysis demonstrated NCTD-induced downregulation of Bcl-2 and Mcl-1 as reported previously but no change in Bcl-xL and Bax.

## Discussion

Malignant gliomas are the most prevalent primary brain tumors in adults with relatively high rates of recurrences. They display a poor response to conventional cytotoxic chemotherapy. Thus, novel approaches to glioma therapy are urgently needed.

The upregulation of the Raf/MEK/ERK cascade is one of the principal Ras-regulated pathways, and has been proven to be associated with glioma cell proliferation, survival, and migration. It has been suggested that downregulation of the Raf/MEK/ERK pathway may be of great promise as a target for preventing tumor cell growth [14]. Therefore, targeting Raf/MEK/ERK pathway is a promising strategy for glioma treatment. Our experiments indicated that both U87 and C6 glioma cells were sensitive to NCTD *in vitro*. NCTD inhibited the Raf/MEK/ERK pathway through downregulation of p-MEK and p-ERK in a concentration-dependent fashion, which is partially in agreement of the previous studies [15]. As the Raf/MEK/ERK pathway is one of the evolutionarily conserved MAPK pathways that play critical roles in driving cell proliferation, survival, and preventing apoptosis, we tested effects of NCTD on glioma cell proliferation and apoptosis. The effect of NCTD on the inhibition of proliferation was measured by MTT assay, and the results proved that NCTD inhibited glioma cell growth in a time- and dose-dependent manner in both U87 and C6 cells. To observe the effect of NCTD on the induction of cell apoptosis, we performed the apoptosis analysis by flow cytometer. Cell apoptosis analysis showed that after treatment with NCTD, both U87 and C6 cells revealed concentration-dependent apoptosis.

One important target for chemotherapy is programmed cell death, and the cell death was determined by the pro-apoptotic and anti-apoptotic proteins. Bcl-2 family proteins play a central role in the control of apoptosis [16]. Antiapoptotic members of Bcl-2 family, including Bcl-2, Bcl-xL as well as Mcl-1 inhibit apoptosis by sequestering Bax. Bax protein is a pro-apoptotic member and the increased expression of this protein often associated with the increased apoptosis in target cells [17]. Cancer cells often express elevated levels of antiapoptotic members of Bcl-2 family, so as to obtain survival and proliferation advantage. Bcl-2 protein is the prototype of this family, which targets intracellular organelles such as the endoplasmic reticulum, outer mitochondrial, and nuclear membranes [18]. Mcl-1 act as an apical molecule in apoptosis control, promoting cell survival by interfering at an early stage in a cascade of events leading to release of cytochrome c from mitochondria. It is reported that Mcl-1 is highly expressed antiapoptotic protein in malignant tumors and cause glioma cell resistance toward apoptosis induced by chemotherapy or radiation therapy [19]. The molecular mechanisms underlying the anticancer activity of NCTD have been extensively studied. Previous studies linked the anticancer activity of NCTD to downregulation of Bcl-2, Bcl-xL and Mcl-1 since it was found that NCTD reduced the level of these antiapoptotic Bcl-2 family proteins in various cancer cells [11,20,21]. In our study, treatment of U87 or C6 cells with NCTD resulted in a significant downregulation of Bcl-2 and Mcl-1. Nevertheless, there were no significant changes in the expression of Bcl-xL or Bax proteins after treatment U87 or C6 cells with NCTD for 24 h. These results suggested that NCTD induced apoptosis in glioma cell lines U87 or C6 through the downregulating of proapoptotic proteins Bcl-2 and Mcl-1.

## Conclusion

In conclusion, we demonstrated that NCTD was an effective potential chemotherapeutic agent for the inhibition proliferation and induction apoptosis of glioma cells. And these results made NCTD a promising therapeutic agent in the treatment of patients with glioma.

## Competing interests

The authors declare that they have no competing interests.

## Authors’ contributions

ZJ and DW designed the study and wrote the article. S-lJ and ZR conducted the experiments and carried out the statistical analyses. S-lG, C-fG, and W-xT assisted with experiments and manuscript preparation. All authors read and approved the final manuscript.
